# Livestock-Associated Methicillin-Resistant *Staphylococcus aureus* (MRSA) in Purulent Subcutaneous Lesions of Farm Rabbits

**DOI:** 10.3390/foods9040439

**Published:** 2020-04-06

**Authors:** Vanessa Silva, Telma de Sousa, Paula Gómez, Carolina Sabença, Madalena Vieira-Pinto, Rosa Capita, Carlos Alonso-Calleja, Carmen Torres, José L. Capelo, Gilberto Igrejas, Patrícia Poeta

**Affiliations:** 1 Functional Genomics and Proteomics Unit, University of Trás-os-Montes and Alto Douro (UTAD), 5000-801 Vila Real, Portugal; vanessasilva@utad.pt (V.S.); telmaslsousa@hotmail.com (T.d.S.); carolinasabenca@hotmail.com (C.S.); gigrejas@utad.pt (G.I.); 2 Department of Genetics and Biotechnology, University of Trás-os-Montes and Alto Douro (UTAD), 5000-801 Vila Real, Portugal; 3 Microbiology and Antibiotic Resistance Team (MicroART), Department of Veterinary Sciences, University of Trás-os-Montes and Alto Douro (UTAD), 5000-801 Vila Real, Portugal; 4 Associate Laboratory for Green Chemistry-LAQV, Chemistry Department, Faculty of Science and Technology, University Nova of Lisbon, 2829-516 Lisbon, Portugal; 5 Area of Biochemistry and Molecular Biology, University of La Rioja, 26006 Logroño, Spain; paula_gv83@hotmail.com (P.G.); carmen.torres@unirioja.es (C.T.); 6 Veterinary and Animal Science Research Center (CECAV), University of Trás-os-Montes and Alto Douro (UTAD), 5000-801 Vila Real, Portugal; mmvpinto@utad.pt; 7 Department of Veterinary Sciences, University of Trás-os-Montes and Alto Douro (UTAD), 5000-801 Vila Real, Portugal; 8 Department of Food Hygiene and Technology, Veterinary Faculty, University of León, E-24071 León, Spain; rosa.capita@unileon.es (R.C.); carlos.alonso.calleja@unileon.es (C.A.-C.); 9 Institute of Food Science and Technology, University of León, E-24071 León, Spain; 10 BIOSCOPE Group, LAQV@REQUIMTE, Chemistry Department, Faculty of Science and Technology, NOVA University of Lisbon, 2825-466 Almada, Portugal; jlcm@fct.unl.pt; 11 Proteomass Scientific Society, 2825-466 Costa de Caparica, Portugal

**Keywords:** MRSA, CC97, rabbit, LA-MRSA, lesions, *Oryctolagus cuniculus*

## Abstract

Methicillin-resistant *Staphylococcus aureus* (MRSA) are one of the main pathogens associated with purulent infections. MRSA clonal complex 97 (CC97) has been identified in a wide diversity of livestock animals. Therefore, we aimed to investigate the antibiotic resistance profiles of MRSA strains isolated from purulent lesions of food-producing rabbits. Samples from purulent lesions of 66 rabbits were collected in a slaughterhouse in Portugal. Samples were seeded onto ORSAB plates with 2 mg/L of oxacillin for MRSA isolation. Susceptibility to antibiotics was tested by the disk diffusion method against 14 antimicrobial agents. The presence of resistance genes, virulence factors and the immune evasion cluster (IEC) system was studied by polymerase chain reaction. All isolates were characterized by multilocus sequence typing (MLST), *agr* and *spa* typing. From the 66 samples analyzed, 16 (24.2%) MRSA were detected. All strains were classified as multidrug-resistant as they were resistant to at least three classes of antibiotics. All isolates showed resistance to penicillin, erythromycin and clindamycin. Seven isolates were resistant to gentamicin and harbored the *aac*(6′)-Ie-*aph* (2″)-Ia gene. Resistance to tetracycline was detected in 10 isolates harboring the *tet*(K) gene. The IEC genes were detected in three isolates. MRSA strains belonged to CC97, CC1, CC5, CC15 or CC22. The isolates were assigned to six different *spa* types. In this study we found a moderate prevalence of multidrug-resistant MRSA strains in food-producing rabbits. This may represent concern for food safety and public health, since cross-contamination may occur, leading to the spread of MRSA and, eventually, the possibility of ingestion of contaminated meat.

## 1. Introduction

*Oryctolagus cuniculus*, commonly known as the European rabbit, is an old species with an enormous ability to adapt to a variety of ecosystems, existing in parallel with—but not dependent on—humans [1,2]. Rabbit meat has excellent dietary and nutritional properties, and is traditional in Mediterranean cuisine [3]. Thus, rabbit farming is a sector of great importance in south-European countries. However, disease outbreaks in the food industry may occur, leading to high economic losses [4]. Slaughterhouses are a key point in monitoring rabbit diseases, as any type of observation and/or information obtained at the slaughterhouse can help to understand and adjust the entire chain [5]. The most frequent lesion found on rabbits’ skin is the purulent lesion, which can be simple or multiple, and of different size and location [6]. Cutaneous or subcutaneous abscesses are common findings in rabbits of all ages. These purulent lesions are usually the result of contamination of dermal wounds and invasion of subcutaneous tissue by pyogenic bacteria, of which the following can be highlighted: *Staphylococcus* (*S*.) spp. (mainly *S. aureus* and *S. hyicus*), *Streptococcus* spp. and *Pasteurella multocida* [7]. Contamination of meat by pathogenic agents is a fundamental point in the area of food safety, since it can compromise the health of consumers. Bacteria use superficial wounds on rabbits’ skin as a route of entry, which is frequent in rabbits kept in cages or in rabbits of considerable size [8,9]. The frequency of multifactorial pathologies in rabbit breeding farms has promoted an excessive use of antibiotics, with the possible presence of residues in meat at dangerous levels and the promotion of antimicrobial resistance in bacteria [10].

One of the main groups of microorganisms that pose a threat to consumers are *S. aureus* strains of animal origin. *S. aureus* are usually part of the normal microbiota of humans and some animal species without causing disease [11]. These bacteria are ubiquitously distributed in nature, and it is possible to isolate them from various food products, being considered one of the most important contaminants in this area [12]. They are adaptable, opportunistic pathogens that have the ability to cause food poisoning due to their ubiquity and ability to form enterotoxins in food, responsible for causing human disease [13]. Besides being able to adapt to various environmental conditions, *S. aureus* have the ability to easily acquire resistance to antimicrobials [14]. Methicillin-resistant *S. aureus* (MRSA) was detected for the first time in 1961 at a hospital in the United Kingdom [15]. MRSA has emerged as a worrisome strain causing a wide variety of illnesses around the world and, although MRSA is primary associated with nosocomial infections, it has been found causing infections in pets, livestock and wild animals [16]. Livestock-associated MRSA (LA-MRSA) often belongs to clonal complex (CC) 398; nevertheless, other MRSA lineages have been identified as associated with LA-MRSA, such as CC1, CC9, CC130, and CC97 [17]. Additionally, livestock have been identified as a reservoir of MRSA, with zoonotic importance [18]. The role of the consumption rabbit as a source of human food makes this species a potential transmitter of genes or resistant bacteria to other animals and even to humans [5]. Therefore, this study aimed to characterize the antimicrobial resistance of MRSA strains isolated from purulent skin lesions of rabbits for consumption, and to determine the genetic lineages of these strains.

## 2. Materials and Methods

### 2.1. Samples and Bacterial Isolates

In a period of four months (from June 2018 to September 2018), 66 pus samples were collected from purulent lesions of rabbits (*Oryctolagus cuniculus*) in a slaughterhouse in the north of Portugal, during the daily tasks related to the sanitary inspection of these animals. One sample was collected from each animal. The samples were kept in refrigeration until further characterization. Samples were placed into liquid Brain Heart Infusion (BHI) with 6.5% NaCl and incubated at 37 °C for 24 h. For *S. aureus* and MRSA isolation, 150 µL of the BHI broth was spread onto Oxacillin Resistance Screening Agar Base (ORSAB) plates supplemented with 2 mg/mL of oxacillin and Mannitol Salt Agar (MSA) plates and incubated at 37 °C for 24 h. One colony from each plate was recovered. *S. aureus* were identified by biochemical tests (Gram staining, DNase and catalase) and by multiplex PCR of the genes 16S rDNA, *nuc* and *mec*A.

### 2.2. Antimicrobial Susceptibility Testing

The phenotypic resistance characterization of the MRSA isolates was performed by the Kirby–Bauer disk diffusion method and according to CLSI 2017 guidelines [19]. Fourteen antibiotics were tested: mupirocin (MUP, 5 μg/disc), tetracycline (TET, 30 μg/disc), trimethoprim-sulfamethoxazole (SXT, 1.25/23.75 μg/disc), fusidic acid (FD, 10 μg/disc), erythromycin (ERY, 15 μg/disc), clindamycin (DA, 2 μg/disc), gentamicin (CN, 10 μg/disc), tobramycin (TOB, 10 μg/disc), penicillin (PEN, 10 U/disc), linezolid (LNZ, 30 μg/disc), cefoxitin (FOX, 30 μg/disc), chloramphenicol (CHL, 30 μg/disc), ciprofloxacin (CIP, 5 μg/disc) and kanamycin (K, 30 μg/disc). *S. aureus* strain ATCC 25,923 was used as quality control in the susceptibility assays.

### 2.3. Antibiotic Resistance Genes and Virulence Factors

The antibiotic resistance genes and virulence factors were determined by PCR with specific primers and conditions as described previously [20,21,22,23]. For the study of resistance to various antibiotics, several resistance genes were tested: *tet*(K), *tet*(M), *tet*(L), *tet*(O), *erm*(A), *erm*(B), *erm*(C), *erm*(T), *msr*(A/B), *mph*C, *lin*A, *lin*B, *vga*A, *vga*B, *vga*C, *vga*E, *cfr*, *aac*(6’)-Ie-*aph*(2´´)-Ia, *aph*(3´)-IIIa, *ant*(4´)-Ia, *ant*(6’)-Ia, *bla*Z, *mup*A, *fus*A, *fus*B and *fusC*. The genes tested for virulence were *hla*, *hlb*, *eta*, *etb tst*, *cna* and Panton–Valentine leucocidin (PVL) (l*uk*F/*luk*S-PV). The presence of the immune evasion cluster (IEC) system was detected by screening for the presence of the *scn* gene (common to all IEC groups), and after by the screening for *chp*, *sak*, *sea* and *sep* genes, determining the IEC group from the combination of these genes [24]. Positive and negative controls from the University of Trás-os-Montes and Alto Douro, Vila Real, Portugal, were used.

### 2.4. Molecular Typing

All MRSA were characterized by *agr*-typing (I–IV) using specific primers [25,26]. The isolates were further characterized by *spa*-typing and multilocus sequence typing (MLST) by PCR and sequencing, and compared to the Ridom SpaServer (http://spaserver.ridom.de) and MLST (https://pubmlst.org/) databases [27,28]. 

## 3. Results

Of the 66 pus samples collected, 50 *S. aureus* (76%) were isolated. Screening for MRSA isolates allowed the identification of 16 (24%) strains, and only these strains were further characterized. MRSA isolates showed resistance to penicillin (*n* = 16), erythromycin (*n* = 16), clindamycin (*n* = 16), cefoxitin (*n* = 14), oxacillin (*n* = 13), ciprofloxacin (*n* = 13), tetracycline (*n* = 10), kanamycin (*n* = 7), gentamicin (*n* = 7), tobramycin (*n* = 7) and fusidic acid (*n* = 3) (Table 1). None of the isolates exhibited resistance to trimethoprim-sulfamethoxazole, linezolid, vancomycin, chloramphenicol or mupirocin. The genotype results for the *nuc*, rDNA 16S and *mec*A genes were positive, allowing us to conclude that all the isolates were methicillin-resistant *S. aureus*, as expected. Among the isolates resistant to macrolides and lincosamides, the majority harbored the *erm*C (*n* = 15) gene, followed by the *erm*B (*n* = 4), *msr*(A/B) (*n* = 2), *vga*B (*n* = 2), *vga*A (*n* = 1) and *lin*B (*n* = 1) genes. The genes conferring resistance to tetracycline were analyzed, and all tetracycline-resistant isolates harbored the *tet*(K) gene and one isolate had both *tet*(K) and *tet*(L) genes. Resistance to aminoglycosides were detected in seven isolates and all were positive to *aac*(6′)-Ie-*aph*(2″)-Ia gene. Regarding the virulence factors, all isolates except one harbored the *hlb* gene, 13 isolates were positive for *cna* and the *eta* gene was detected in only one isolate. All isolates were PVL-negative and the *scn* gene was detected in three isolates which were ascribed to type B and C. All isolates were *agr* type III. MLST analysis showed that the most common ST was ST2855, detected in 50% of the isolates, and categorized as clonal complex CC97. The remaining isolates were ascribed to five sequence types (ST105, ST582, ST5, ST22 and ST1) included in clonal complexes CC5, CC15, CC22 and CC1. Among the 16 MRSA isolates, six different *spa*-types were detected (t1190, t002, t2802, t084, t032 and t1491), with *spa*-types t1190 and t2802 being the predominant ones.

## 4. Discussion

Dermatitis and purulent lesions are the main prominent *S. aureus* infections in rabbits [29]. Purulent subcutaneous lesions (i.e., abscesses) are relevant due to the technical impact they present in slaughterhouses, as well as in the economic sector and animal welfare. In this study, MRSA strains were found in 24% of samples analyzed, which is in agreement with other studies that reported the presence of *S. aureus* and MRSA in purulent lesions of farm rabbits [30,31]. Given the resistance to three or more different antibiotic classes found in all isolates, all MRSA were classified as multidrug-resistant.

### 4.1. Characterization of CC97 MRSA Isolates

The great majority of the isolates were ascribed to ST2855, which is included in clonal complex CC97. This particular ST was only reported in two recent studies, conducted in Portugal and Spain, in wild hares and farm rabbits [31,32]. These results suggest that ST2855 may either be associated with lagomorphs or be endemic of the Iberian peninsula [32]. The study conducted with farm animals evaluated the presence of MRSA in rabbit lesions. However, unlike our study, no MRSA was found in abscesses [31]. Although CC97 has been previously identified in both community and LA-MRSA, this clonal complex is frequently associated with livestock, particularly ruminants and pigs [33]. Moreover, CC97 is one of the major MRSA clonal complexes in bovines and a leading cause of bovine mastitis worldwide [34]. In Portugal, the presence of this clone has been reported among healthy bovines [35]. CC97 isolates belonged to either *spa*-type t1190 or t2802. *spa*-type t1190 is a rabbit-specific strain since it has been previously found in rabbit meat [36,37] and lesions [38]. As for *spa*-type t2802, it has been reported mostly associated with bovine livestock but also with humans [39,40]. All CC97 isolates lacked the IEC genes associated with human adaptation, clearly pointing to an animal origin. Furthermore, five of the eight CC97 MRSAs showed resistance to tetracycline, conferred by the *tet*(K) gene, which, along with IEC-negative and based on epidemiological evidence, is an indication of animal origin [41]. As in our study, in other studies reporting the presence of CC97 MRSA, the isolates are frequently multidrug resistant with resistance only to β-lactams and tetracyclines but also to macrolides, lincosamides and aminoglycosides [17,31]. Although the relationship between antibiotic resistance profiles and clonal complexes is still not clear, some studies suggest that different LA-MRSA clones may present specific resistance patterns [42]. Macrolide-lincosamide-streptogramin (MLS) resistance occurs by the cross-resistance among these antibiotics, and bacteria with resistance to MLS were previously reported to be transmitted from animals to humans through the food chain [43]. Resistance to macrolides and lincosamides was detected in all CC97 MRSA isolates. Although it is assumed that *erm*(A) or *erm*(C) genes predominate in *S. aureus*, *erm*(C) was the most prevalent in this study. The *erm* genes are highly associated with MLS resistance since these genes cause a 23S rRNA methylation leading to a ribosomal alteration [44]. Three CC97 isolates also presented resistance to gentamicin and harbored the *aac*(6′)-Ie-*aph*(2″)-Ia gene. The accessory gene regulator (*agr*) system of *S. aureus* regulates numerous virulence and pathogenicity factors. CC97 MRSA strains have been repeatedly typed as *agr* type I in pigs and bovine isolates [17,45,46]. Nevertheless, in our study all CC97 isolates belonged to *agr* type III, which may be characteristic of ST2855 since in the only study reporting ST2855 in rabbits, all ST2855 isolates from farm rabbit infections were also typed as *agr* III [31]. Studies have tried to find a relationship between the type of *agr* and the type of infections and toxins produced by *S. aureus* and, although *agr* III MRSA isolates are often *tst*-positive, in our study the *tst* gene was not identified [47,48].

### 4.2. Characterization of Non-CC97 MRSA Isolates

Regarding the non-CC97 MRSA isolates, they were typed into CC1, CC5, CC15 and CC22. Only one isolate was attributed to CC1, which is a clone that seems to have a low host specificity. CC1 has been identified in human infections as part of community transmission, but it has also been isolated from animals, namely, horses, bovines and pigs [33,49]. The CC1 isolate was typed as *spa*-type t1491, which is generally associated with methicillin-susceptible *S. aureus* (MSSA) and has been identified in pigs and humans [50,51]. One isolate belonged to CC15 and *spa*-type t084. CC15 has been widely described in the literature, but this clone is typically linked with MSSA [52]. As for *spa* t084, it has been described as being associated with this clonal complex [53]. The remaining isolates belonged to the classical human MRSA lineages CC2 and CC5. The CC15 and two CC5 isolates also contained the IEC genes and were typed as IEC-B and -C, respectively. The presence of IEC genes suggests a human origin and possibly a human contamination, which is not very surprising since these clones are usually associated with human infections [54]. Furthermore, the detection of IEC-positive MRSA strains out of the human niche is rare [55]. As with MRSA ST398 often originating in pigs, in which most isolates are resistant to tetracycline, in our study a total of 10 (out of 16) also presented resistance to tetracycline [56]. Out of the eight non-CC97 isolates, five showed resistance to tetracycline conferred by the *tet*(K) gene and one isolate also harbored *tet*(L). Resistance to tetracycline can be acquired by *tet*(M) and *tet*(O) genes which are located in transposon or chromosome and/or by *tet*(K) and *tet*(L) genes located in plasmids [57]. Some of these plasmids carried additional resistance genes, such as the macrolide resistance gene *erm*(B) [58]. Unlike CC97-MRSA isolates, which harbored only the *erm*(C) gene conferring resistance to macrolides, the non-CC97 isolates also hosted the *erm*(B) (*n* = 4) and *msr*(A/B) (*n* = 2).

Farm animals are often in contact with bacteria present in the environment where they are raised, thus increasing the likelihood of genetic information exchange between animal and environmental or even human bacteria, allowing commensal bacteria to gain certain genes that give them resistance to several antibiotics [59,60]. The overuse of antibiotics in the food-producing animal sector also contributes to antibiotic resistance. It is also important to point out that, in Portugal, the main antibiotics used in cuniculture are tetracyclines and polymyxins. In this study, more than half of isolates showed resistance to tetracycline, which may reflect the overuse of this class of antibiotics in consumption rabbits. These resistances can later be transferred directly to the human microbiome [61]. Antibiotics are often provided to food-producing animals without prior culture and antibiotic susceptibility testing, which makes these animals an ideal reservoir for multidrug-resistant bacteria [62]. Additionally, animals arriving at slaughterhouses often carry *S. aureus* strains, not only causing infection but also as asymptomatic colonizers. 

## 5. Conclusions

Food-producing animals, particularly rabbits, may be a reservoir of multidrug-resistant MRSA strains that can be considered an important issue in terms of food safety. The prevalence of bacteria resistant to antibiotics often used in human medicine is alarming. MRSA are increasingly affecting the animal world. New types of MRSA are emerging in several species of animals, and this may become a threat to human health through occupational exposure. In this study, the rate of MRSA strains found infecting rabbits is a concern, since the transmission of MRSA strains from rabbit to human or between rabbits may occur. Moreover, some of our MRSA strains had a human origin and we can hypothesize that animal handlers and farmers might be a source of contamination and transmission of these strains. This study allows veterinarians and those responsible for the farms to have a better understanding of the possible causes of the occurrence of these purulent lesions in animals, to treat them more effectively through appropriate antibiotic use and to be more cautious in terms of biosafety in slaughterhouses.

## Figures and Tables

**Table 1:** Genetic characterization of antibiotic resistance and molecular typing of methicillin-resistant *Staphylococcus aureus* (MRSA) isolates from rabbits. CN: gentamicin; DA: clindamycin; CIP: ciprofloxacin; ERY: erythromycin; FD: fusidic acid; FOX: cefoxitin; IEC: immune evasion cluster; PEN: penicillin; PVL: Panton–Valentine leucocidin; TET: tetracycline; TOB: tobramycin.

Isolates	Resistance		Virulence Factors		Molecular Characterization		
	Phenotype	Genotype	IEC Type	Other Genes	ST/CC	*spa*-type	*agr*-type
**VS2745**	FOX, PEN, TET, ERY, DA, CIP	*mec*A, *tet*(K), *erm*(C), *vga*B	*-*	*hlB, cna*	ST2855/CC97	t1190	III
**VS2746**	FOX, PEN, TET, ERY, DA	*mec*A, *tet*(K), *erm*(C)	*-*	*hlB*	ST2855/CC97	t1190	III
**VS2747**	FOX, PEN, ERY, DA, CIP	*mec*A, *erm*(C)	*-*	*hlB, cna*	ST2855/CC97	t1190	III
**VS2748**	FOX, PEN, TET, ERY, DA, CN, TOB, CIP	*mec*A, *tet*(K), *erm*(B), *erm*(C)*, aac*(6′)-Ie-*aph*(2″)-Ia	B	*hlB, cna*	ST105/CC5	t002	III
**VS2749**	FOX, PEN, TET, ERY, DA, CIP	*mec*A, *tet*(K), *erm*(C)	*-*	*hlB, cna*	ST2855/CC97	t1190	III
**VS2750**	FOX, PEN, TET, ERY, DA, CN, TOB, CIP	*mec*A, *tet*(K), *erm*(C)*, aac*(6′)-Ie-*aph*(2″)-Ia	*-*	*hlB, cna*	ST2855/CC97	t2802	III
**VS2751**	FOX, PEN, TET, ERY, DA, CN, TOB, CIP	*mec*A, *tet*(K), *erm*(C)*, aac*(6′)-Ie-*aph*(2″)-Ia	*-*	*hlB*	ST2855/CC97	t2802	III
**VS2752**	FOX, PEN, TET, ERY, DA, CN, TOB, CIP	*mec*A, *tet*(K), *erm*(C)*, aac*(6′)-Ie-*aph*(2″)-Ia	*-*	*hlB, cna*	ST5/CC5	t002	III
**VS2753**	FOX, PEN, ERY, DA, TOB	*mec*A, *erm*(C)*, vga*A	*-*	*hlB, cna*	ST2855/CC97	t2802	III
**VS2754**	FOX, PEN, TET, ERY, DA, CN, CIP	*mec*A, *tet*(K), *erm*(C)*, aac*(6′)-Ie-*aph*(2″)-Ia	*-*	*hlB, cna*	ST5/CC5	t1094	III
**VS2755**	FOX, PEN, ERY, DA, FD	*mec*A, *erm*(B), *erm*(C)	*-*	*hlB, cna*	ST5/CC5	t002	III
**VS2756**	FOX, PEN, ERY, DA, CIP, FD	*mec*A, *erm*(B), *erm*(C)*, msr*(A/B)	C	*hlB, etA, cna*	ST582/CC15	t084	III
**VS2757**	FOX, PEN, ERY, DA, CIP, FD,	*mec*A, *erm*(C)	B	*hlB, cna*	ST5/CC5	t002	III
**VS2758**	FOX, PEN, ERY, DA, CN, TOB, CIP	*mec*A, *erm*(C), *aac*(6′)-Ie-*aph*(2″)-Ia	*-*	*hlB*	ST2855/CC97	t2802	III
**VS2759**	FOX, PEN, TET, ERY, DA, CIP, FD	*mec*A, *tet*(K), *tet*(L), *erm*(B)*, msr*(A/B), *lin*B	*-*	*cna*	ST22/CC22	t032	III
**VS2760**	FOX, PEN, TET, ERY, DA, CN, TOB, CIP	*mec*A, *tet*(K), *erm*(C)*, vga*B, *aac*(6′)-Ie-*aph*(2″)-Ia	*-*	*hlB, cna*	ST1/CC1	t1491	III
